# Systemic Amyloidosis Presenting as Budd-Chiari Syndrome: A Case Report

**DOI:** 10.34172/mejdd.2024.391

**Published:** 2024-07-31

**Authors:** Naman Lodha, Samarth Bhat K S, Kartikeya Mathur, Vikrant Verma, Rengarajan Rajagopal, Chhagan Lal Birda, Ashish Agarwal

**Affiliations:** ^1^Department of General Medicine, All India Institute of Medical Sciences, Jodhpur, Rajasthan, India; ^2^Department of Gastroenterology, All India Institute of Medical Sciences, Jodhpur, Rajasthan, India; ^3^Department of Pathology, All India Institute of Medical Sciences, Jodhpur, Rajasthan, India; ^4^Department of Diagnostic and Interventional Radiology, All India Institute of Medical Sciences, Jodhpur, Rajasthan, India

**Keywords:** Budd-Chiari syndrome, Systemic amyloidosis, Hypercoagulability, Infiltrative liver disease

## Abstract

Budd-Chiari syndrome (BCS) is characterized by hepatic venous outflow tract obstruction and is commonly associated with an underlying hypercoagulable state. Systemic amyloidosis is a disorder characterized by systemic deposition of misfolded proteins leading to end organ damage. Amyloidosis is commonly associated with coagulation abnormalities, mainly leading to increased bleeding diathesis. Here, we report a case of amyloid light chain (AL) amyloidosis presenting as BCS. A 40-year-old man presented with abdominal distension along with anorexia and weight loss. On evaluation, he had severe hypoalbuminemia, raised alkaline phosphatase, and non-visualization of hepatic veins on abdominal imaging. Further evaluation confirmed the diagnosis of AL amyloidosis with renal, cardiac, and hepatic involvement. AL amyloidosis rarely can present with BCS. A high index of suspicion is needed as symptoms can be variable and non-specific.

## Introduction

 Budd-Chiari syndrome (BCS) is a disorder that involves obstruction of the hepatic venous outflow tract, provided the obstruction is not due to cardiac disease, pericardial disease, or sinusoidal obstruction syndrome.^1^ An underlying prothrombotic disorder can be identified in over 75% of the patients, with myeloproliferative disorders being the most common. Other causes include congenital hypercoagulable disorders, infections, pregnancy, and malignancy.^1^ Amyloidosis is also a rare disease and is characterized by extracellular tissue deposition of unfolded proteins in various organs such as the kidney, heart, and gastrointestinal system, leading to organ dysfunction. Amyloidosis is generally associated with increased bleeding tendency and is associated with one or more of several causes, including reduced activity of factor X, vascular infiltration with amyloid, and abnormal liver function due to amyloid deposition. Bleeding due to acquired von Willebrand disease or factor IX deficiency has also been described in primary amyloidosis.^1–4^ Amyloidosis is rarely reported as a cause of BCS. We report a unique case of BCS, which, on evaluation, uncovered a diagnosis of underlying systemic AL amyloidosis.

## Case Report

**Figure 1:**
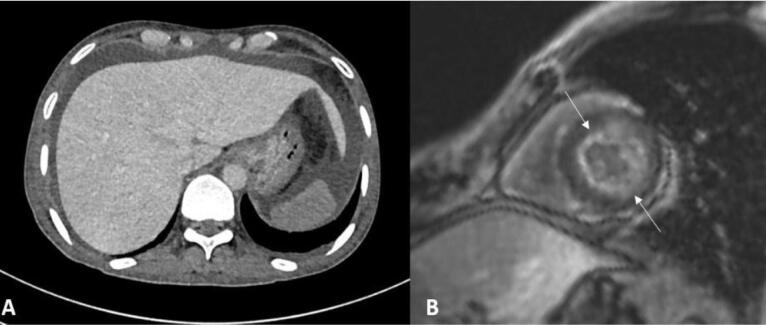
(A) tomogram (hepatic venous phase-axial section) showing hepatomegaly with heterogeneous enhancement pattern and ascites with non-visualization of hepatic veins. (B). Short axis section at mid-ventricular level - inversion recovery sequence showing diffuse subendocardial enhancement (marked by white arrows)

 A 40-year-old man presented with a 4-month history of progressive pedal edema and abdominal distension. He reported constitutional symptoms, including anorexia and a significant weight loss of 20 kg. No other significant personal or family history was present. On examination, bilateral pitting pedal edema and shifting dullness were noted. Hemogram and renal function tests were within normal limits, but liver function tests revealed elevated alkaline phosphatase levels (2484 IU/L), low total protein (3.0 g/L), and low serum albumin (1.0 g/dL) levels. The lipid profile showed elevated serum cholesterol (535 mg/dL) and low-density lipoprotein levels (414 mg/dL), while the thyroid profile revealed an elevated thyroid-stimulating hormone (TSH) (22.1 mIU/mL) with normal T3 and T4 levels. An ultrasound followed by a triple-phase contrast-enhanced computed tomography of the abdomen revealed non-visualization of all three hepatic veins suggestive of Budd-Chiari syndrome, with the presence of gross ascites (Figure 1A). An ascitic tap was done, which was suggestive of high serum ascites albumin gradient (SAAG) and low protein ascites. The patient was started on low molecular weight heparin followed by dabigatran. Upper gastrointestinal endoscopy was done to rule out esophageal varices, which was normal. An etiological workup for common thrombophilic conditions such as myeloproliferative neoplasm, factor 5 Leiden mutation, serum homocysteine, protein C and S, anti-thrombin III deficiency, anti-phospholipid antibody syndrome profile, and Janus kinase (JAK 2) mutation was negative. A routine urine examination showed 3 + proteinuria, followed by a 24-hour urine protein assessment, which showed nephrotic range proteinuria (5.1 g/24 hours).

**Figure 2:**
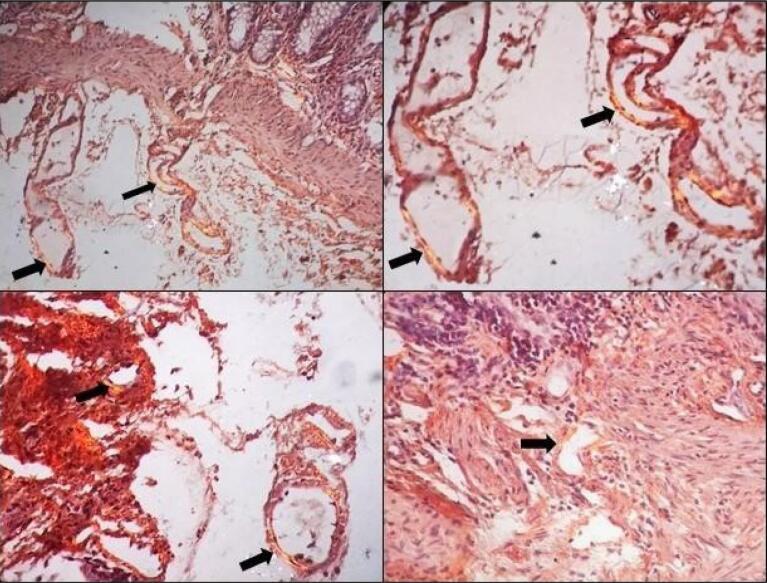
Vessels showing apple-green birefringence under polarized microscopy (arrows)

 Given the multisystem involvement (renal & hepatic) with raised alkaline phosphatase level, a possibility of infiltrative liver disorder, like amyloidosis, was considered. Abdominal fat pad aspiration cytology was negative for amyloidosis, but rectal biopsy showed deposition of hyaline and eosinophilic, amorphous material in the vessel walls. These deposits were congophilic and showed apple-green birefringence under a polarized microscope, suggestive of amyloidosis (Figure 2). Immunoperoxidase staining for serum amyloid A (SAA) associated protein was negative. Serum protein electrophoresis with immunofixation was normal with an absence of monoclonal protein spike. The radiographic skeletal bone survey did not reveal the presence of lytic lesions. A bone marrow core biopsy was essentially normal, with an absence of evidence of multiple myeloma. Serum free light chain assay was suggestive of lambda > kappa light chain elevation with kappa/lambda ratio of 0.145 suggestive of light chain disease. A cardiac magnetic resonance imaging (MRI) was done to evaluate for cardiac involvement with amyloidosis, and it showed concentric left ventricle hypertrophy with diffuse subendocardial enhancement in the left ventricle and right atrium with thickened interatrial septum, consistent with cardiac amyloidosis (Figure 1B).

 Thus, a diagnosis of AL type of systemic amyloidosis with renal and cardiac involvement with Budd Chiari syndrome was confirmed. All the investigations are summarised in Table 1.

**Table 1:** Baseline investigations

**Investigation**	**Findings**
Hemoglobin	12.6 g/dL (13-16 g/dL)
Total leucocyte count	5.18 cells/uL (4000-11000/uL)
Platelet count	393 x 10^3^/uL (150 × 10^3^ – 450 × 10^3^/uL)
Urea	19 mg/dL (6-24 mg/dL)
Creatinine	0.62 mg/dl (0.7-1.4 mg/dL)
Bilirubin – total	0.52 mg/dL (0.3-1.2 mg/dL)
Bilirubin- conjugated	0.20 mg/dl ( < 0.2 mg/dL)
Aspartate transaminases (AST)	35.2 IU/L (8-33 IU/L)
Alanine transaminases (ALT)	20.4 IU/L (8-48 IU/L)
Alkaline phosphatase (ALP)	2484 IU/L (30-120 IU/L)
Total protein	3.05 g/dL (6.6 -8.3 g/dL)
Serum albumin	1.03 g/dL (3.5-5.2 g/dL)
24-hour urinary protein	5168 mg/d ( < 100 mg/d)
Urine ACR	3742 mg/g (30-299 mg/gm)
TSH	24.37 mIU/L (0.3-3.6 mIU/L)
ESR	79 mm/h (0-20 mm/h)
GGT	1207 U/l (male < 55 U/L)
Urine microscopy	Appearance – Pale yellow clear PH: 6.5 Specific gravity: 1.010 Protein: + + + Ketone: Nil Sugar: Nil Bilirubin: Nil Microscopy: - Epithelial cells- occasional Pus cells-occasional RBCs: - occasional
Lipid profile	Serum cholesterol 432 mg/dL (High > 240 mg/dL) Serum HDL 76 mg/dL (High > 60 mg/dL) Serum LDL 301 mg/dL (High > 160 mg/dL) Serum triglyceride 236 mg/dL (High > 200 mg/dL)
APLA profile	Anti-beta-2-GP1 IgG - 10.25 RU/mL (Negative < 20.0 RU/mL) Anti-beta-2-GP1 IgM - 11.44 RU/mL (Negative < 20.0 RU/mL) Anti-cardiolipin IgG - 2.18 U/mL (Negative: < 12.0 U/mL) Anti- cardiolipin IgM - 3.96 U/mL (Negative: < 12.0 U/mL) LA1/LA2 ratio: 0.92 (Normal < 1.20) Lupus anticoagulant is negative
ANA	Negative
Homocysteine	2.91 μM/L (3.7–13.9 μM/L)
Activated protein C resistance assay	PCAT – 129 s (128–173 s) PCAT/O – 85.1 sec (68–91 s)
Anti-thrombin	90 % (80–120)
PT/INR	13.6/1.01
CECT triple phase abdomen	The liver is enlarged in size (18.5 cm). Both lobes of the liver show patchy heterogenous parenchymal enhancement. A hemangioma measuring 3 × 2.5 cm is seen in segment V/VII of the liver. All three hepatic veins are not opacified in the hepatic venous/delayed phase. The hepatic segment of IVC appears narrowed. On USG correlation, the right hepatic vein is not visualized/thrombosed, the distal segment of the middle hepatic vein appears narrowed, and stenosis of the left hepatic vein is seen at ostia. No IHBR dilatation is noted in both lobes of the liver. MPV measures 12.6mm. The splenic vein is narrowed in caliber with partial thrombosis. Hepatomegaly with heterogenous parenchymal enhancement. Hemangioma in segment V/VII. Hypoperfusion complex. Heterogeneously enhancing mediastinal lymph nodes in visualized sections.
MRI cardiac	Mildly dilated RA Mildly dilated LA Inter-atrial septum measures ~ 5.1 mm in maximum thickness Concentric LV hypertrophy is seen. No regional wall motion abnormality is seen. Diffuse subendocardial LGE is seen in entire LV, RV, RA and LA Impression: Features are consistent with amyloidosis
Bone marrow aspiration & trephine biopsy	Bone marrow biopsy is in a single linear core with 8-9 partly preserved intertrabecular spaces wherein the marrow is cellular for age, showing all hematopoietic components. No increase in plasma cells or deposits of amorphous material was seen. There is no evidence of a cluster of plasma cells or deposits of amorphous material. Impression: Cellular marrow reveals all hematopoietic components
Abdominal fat pad biopsy	No evidence of amyloid deposition was seen.
Rectal biopsy	Microscopic examination: The section examined shows fragments of rectal mucosa displaying largely maintained mucosal architecture. The lamina propria shows moderate inflammatory infiltrate comprising lymphocytes and few plasma cells, along with few lymphoid aggregates. A few of the small vessels show mild deposition of hyaline and eosinophilic amorphous deposits. These deposits are congophilic and show apple-green birefringence under polarized microscopy Immunohistochemistry for Serum Amyloid A (SAA) was non-contributory Impression: Consistent with amyloidosis
Serum free light chain assay	Free kappa light chain-52 mg/L (2.37-20.73)
	Free lambda light chain-358.5mg/L (4.23 27.69)
	Kappa/lambda ratio- 0.145 (0.31-1.56)

Abbreviations: ALT: alanine transaminases; AST: aspartate transaminases; ESR: Erythrocyte Sedimentation rate; ACR: Albumin Creatinine Ratio; PT: Prothrombin time; INR: International Normalised Ratio; PCAT: Protein C Activation time.

 The patient was planned to receive a bortezomib-based chemotherapy-based regimen, but he started developing high-grade fever with shortness of breath. He later developed sepsis with septic shock and succumbed to illness.

## Discussion

 BCS is an uncommon disease that is characterized by blockage of hepatic veins leading to obstruction of the hepatic venous outflow tract.^1^ Most patients with BCS have an underlying prothrombotic disorder, with myeloproliferative disorders being the most common cause. Nephrotic syndrome, as a result of loss of hemostatic proteins in the urine, is a prothrombotic state and is associated with a higher incidence of both arterial and venous thrombosis, mainly deep veins and renal vein thrombosis.^2^ However, BCS in patients with nephrotic syndrome is rare, and BCS in a patient with nephrotic syndrome due to systemic immunoglobulin light chain (AL) amyloidosis is extremely rare, with only a few case reports describing such an association.^2-4^

 AL amyloidosis is a disease that occurs in elderly patients and results from a systemic deposition of fragments of monoclonal light chain, leading to organ dysfunction. Patients with amyloidosis often have coagulation abnormalities, with bleeding diathesis being a recognized manifestation. The increased bleeding tendency is mainly a result of Factor X deficiency, with the deficiency of other clotting factors also described.^5^ Further, amyloid infiltration of blood vessels and enhanced fibrinolytic activity also play a role in increased bleeding tendencies of patients with amyloidosis.^6^ Although less commonly recognized, some studies have also described increased thrombotic tendencies in patients with amyloidosis, which has been ascribed to the impairment of the thrombin-antithrombin pathway in association with low anti-thrombin biological activity.^7^ However, hepatic venous thrombosis leading to BCS is an extremely rare association. Notably, our case is further unique, as our patient presented in the 4^th^ decade of life, whereas less than one percent of cases of amyloidosis occur in this age group.

 Although the patient was initially planned for a trans-jugular intrahepatic portosystemic shunt (TIPSS) procedure for BCS, he was eventually found to have systemic amyloidosis with renal, gastrointestinal, and cardiac involvement. The case highlights the importance of considering a wide range of differential diagnoses, even when the presentation is typical for a certain condition, as was the case with BCS in our patient. The diagnosis of amyloidosis was uncovered only when a urinary examination for proteinuria was done for hypoalbuminemia, which was initially ascribed to hepatic dysfunction due to BCS. This emphasizes the crucial role of urinary evaluation in patients with chronic liver disease, where hypoalbuminemia is commonly attributed to the underlying liver disease.

 In conclusion, physicians should thus be aware of the diverse clinical presentations of amyloidosis and consider performing a urinary examination in patients with significantly low albumin, including those with underlying liver disorders, as it may uncover other diseases, such as amyloidosis in our case.
